# POEMS syndrome presenting with *Vibrio vulnificus*–like cutaneous lesions: a CARE guidelines-compliant case report

**DOI:** 10.3389/fmed.2025.1625877

**Published:** 2025-07-08

**Authors:** Xiaomei Li, Bihong Lin, Liping Lin, Conghua Song

**Affiliations:** ^1^School of Basic Medicine, Putian University, Putian, Fujian, China; ^2^Key Laboratory of Translational Tumor Medicine in Fujian Province, Putian University, Putian, Fujian, China; ^3^Department of Gastroenterology, The Affiliated Hospital of Putian University, Putian, Fujian, China; ^4^Gastrointestinal Endoscopy Center, The Affiliated Hospital of Putian University, Putian, Fujian, China

**Keywords:** POEMS syndrome, cutaneous manifestations, monoclonal plasma cell disorder, *Vibrio vulnificus*, case report

## Abstract

**Background:**

POEMS (Polyneuropathy, Organomegaly, Endocrinopathy, Monoclonal plasma cell disorder, Skin changes) syndrome is a rare paraneoplastic disorder driven by a λ-restricted plasma cell clone. Cutaneous manifestations are common but typically limited to hyperpigmentation, hemangiomas, and sclerodermoid changes. We herein report the first case of POEMS syndrome presenting with skin lesions closely resembling those of *Vibrio vulnificus* infection.

**Case presentation:**

A 65-year-old woman was admitted with malignant hypertension, refractory ascites, symmetrical thigh ecchymoses evolving into tense hemorrhagic bullae, and progressive sensorimotor polyneuropathy. Laboratory workup revealed thrombocytosis, hypoalbuminemia, acute kidney injury, an IgA-λ monoclonal band on serum immunofixation, and a markedly elevated VEGF level (729.7 pg./mL, reference range: 9–86 pg./mL). Electromyography confirmed a demyelinating neuropathy. Infectious, autoimmune, hepatic, renal, and malignant etiologies were systematically excluded. A multidisciplinary team reached the diagnosis of POEMS syndrome based on two mandatory criteria (polyneuropathy and monoclonal plasma cell disorder), one major criterion (elevated VEGF), and multiple minor criteria (extravascular volume overload, endocrinopathy, skin changes, thrombocytosis, organomegaly).

**Management and outcome:**

The patient received bortezomib plus dexamethasone, leading to gradual resolution of skin lesions, improvement of neuropathic symptoms, and reduction of VEGF levels. After six cycles, ascites resolved and neurological function partially recovered.

**Conclusion:**

This case expands the spectrum of POEMS-associated skin changes to include *V. vulnificus*–like bullous lesions. “*V. vulnificus*–like” refers purely to morphological similarity and not microbiological confirmation. Awareness of such atypical presentations is essential for early recognition. Clinicians should consider POEMS syndrome in patients with unexplained multisystem involvement and vascular skin changes. VEGF measurement and immunofixation electrophoresis are essential tools for timely diagnosis.

## Introduction

POEMS syndrome is a rare multisystemic paraneoplastic disorder caused by an underlying monoclonal plasma cell proliferative disease, most often involving *λ* light chain–restricted clones (1). The acronym “POEMS” reflects its hallmark features: Polyneuropathy, Organomegaly, Endocrinopathy, Monoclonal plasma cell disorder, and Skin changes. First described in the 1980s, POEMS syndrome remains underdiagnosed due to its heterogeneous and often insidious clinical presentation. In addition to the core features, patients frequently exhibit extravascular volume overload (ascites, pleural effusion), papilledema, thrombocytosis or polycythemia, and markedly elevated levels of vascular endothelial growth factor (VEGF), which plays a pivotal role in the disease pathogenesis and correlates with disease activity and symptom severity.

Cutaneous manifestations are present in over 70% of POEMS patients and can serve as important diagnostic clues (2). Common dermatologic findings include hyperpigmentation, hypertrichosis, hemangiomas (particularly glomeruloid or cherry-type), acrocyanosis, and sclerodermoid skin thickening. However, bullous or necrotic lesions resembling infectious processes are exceedingly rare and may lead to diagnostic confusion. Notably, the occurrence of hemorrhagic bullae mimicking *Vibrio vulnificus* infection has not been previously reported in the context of POEMS syndrome.

Herein, we describe a unique case of POEMS syndrome in a 65-year-old woman who presented with rapidly progressive violaceous ecchymoses and hemorrhagic bullae on the lower limbs, closely mimicking *V. vulnificus*–associated cellulitis. “*Vibrio vulnificus*-like” refers purely to morphological similarity and not microbiological confirmation. This atypical dermatologic presentation, combined with systemic features and an elevated VEGF level, ultimately led to a unifying diagnosis. To our knowledge, this is the first reported case of POEMS syndrome manifesting with *V. vulnificus*–like cutaneous changes. This case highlights the need for heightened clinical suspicion in patients with multisystem involvement and unusual vascular skin lesions. This case report adheres to the CARE guidelines to ensure completeness, transparency, and ethical compliance in reporting clinical details.

## Case description

A 65-year-old woman presented with progressive bilateral lower limb purpuric plaques and edema for 1 month. The skin lesions were violaceous, tender, and edematous, measuring approximately 18 × 7.5 cm on the thighs, initially raising suspicion for *V. vulnificus* infection. She denied fever, trauma, or exposure to seawater or seafood. Over the past 7 months, she had developed generalized edema, ascites, exertional dyspnea, and proteinuria, and experienced intermittent dizziness for the last 2 years. Her history included schizophrenia managed with risperidone. There was no relevant family or autoimmune history.

On examination, she was cachectic, with striking thigh purpura, skin hyperpigmentation, bilateral pitting edema, and abdominal distension. Neurological findings included symmetrical distal muscle weakness, sensory deficits, and absent tendon reflexes. Laboratory tests revealed thrombocytosis (527 × 10⁹/L), hypoalbuminemia, renal insufficiency, and elevated inflammatory markers. Thyroid function showed persistent hypothyroidism despite treatment. Ascitic fluid was transudative and sterile. Imaging revealed hepatosplenomegaly and pelvic CT showed irregular sacroiliac joint margins and mild joint space narrowing without lytic lesions. Ophthalmoscopy was unremarkable.

**Figure 1:**
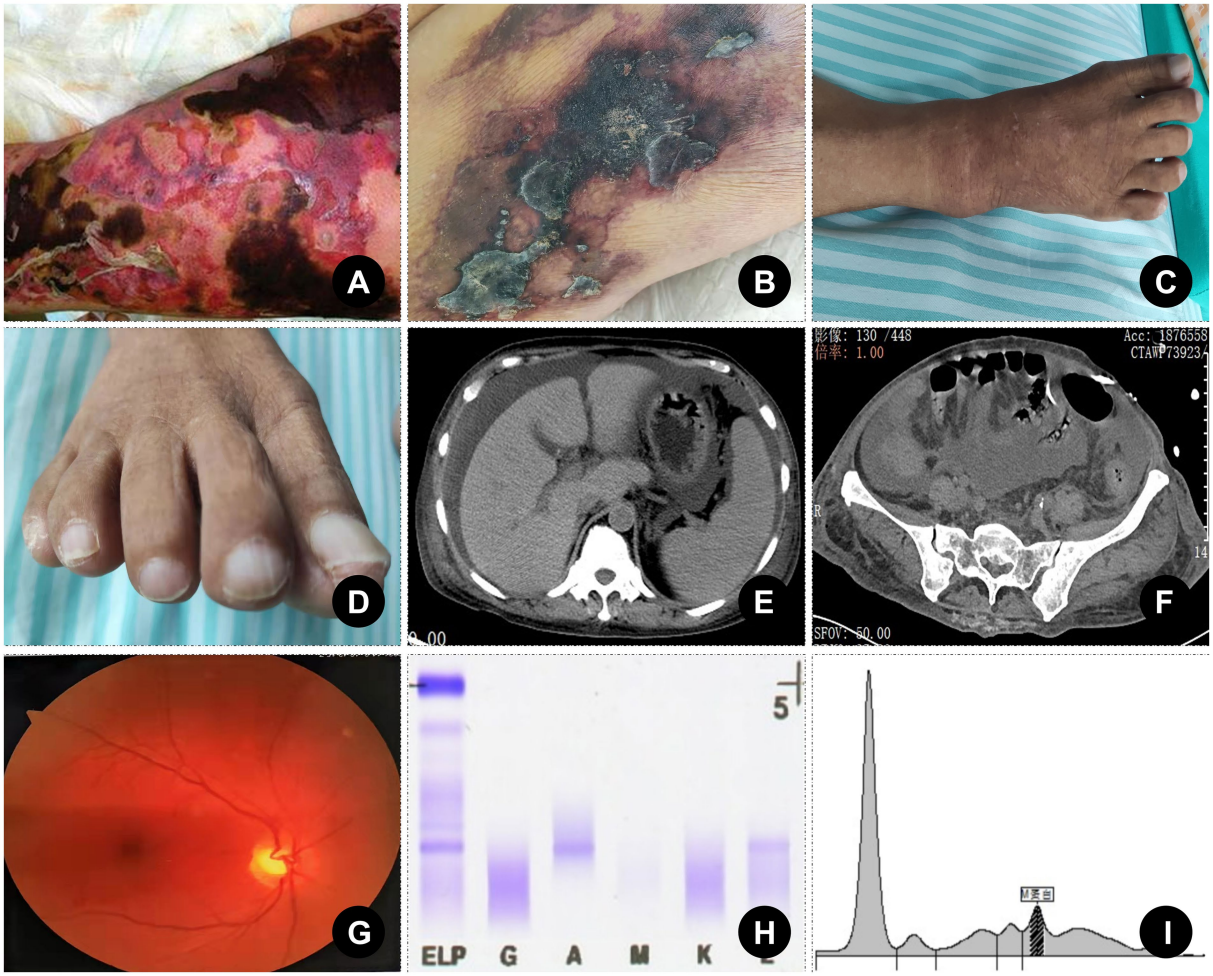
Skin changes and key diagnostic evidence. **(A)** Skin changes in the lower limbs caused by *Vibrio vulnificus* infection (the past real case at our center with written informed consent obtained). **(B)** Skin changes in the lower limbs of the case. **(C)** Pigmentation of lower limb skin and whitening of toenails (distant view). **(D)** Pigmentation of lower limb skin and whitening of toenails (close view). **(E)** A cross-sectional CT scan suggested ascites and splenomegaly. **(F)** Pelvic CT showed irregular sacroiliac joint margins and mild joint space narrowing without lytic lesions. **(G)** Fundus examination was unremarkable. **(H)** Elevated serum VEGF (729.71 pg./mL, reference range: 9–86 pg./mL). **(I)** Monoclonal IgA-λ protein on serum immunofixation electrophoresis.

Given the constellation of symptoms, POEMS syndrome was considered. Further investigations revealed demyelinating polyneuropathy on electromyography, elevated serum VEGF (729.71 pg./mL), and monoclonal IgA-λ protein on serum immunofixation electrophoresis (SIFE). The serum free light chain *κ*/λ ratio was markedly reduced (0.262). Urine immunofixation was negative. The key indicators are shown in Figure 1. Despite family refusal for bone marrow, lymph node, and sural nerve biopsies, a multidisciplinary consultation supported the diagnosis of POEMS syndrome based on the 2023 updated criteria (3). The unusual skin lesions, initially suggestive of *V. vulnificus* infection, were ultimately recognized as paraneoplastic manifestations of POEMS—a rare but important dermatologic clue.

## Timeline

The patient first presented in February 2024 with hypertensive emergency and marked edema, followed by recurrent proteinuria, renal dysfunction and motor dysfunction of both lower extremities. At the initial presentation, the patient already exhibited symmetric lower limb muscle weakness and sensory deficits, which progressed over time. In September 2024, she was hospitalized due to worsening abdominal distension and lower limb ecchymosis. After extensive evaluations-including liver and kidney function tests, ascitic fluid analysis, tumor markers, thyroid function, infectious and autoimmune screenings—common causes of ascites were ruled out. With the development of blistering and exfoliative skin lesions, a systemic review revealed features such as neuropathy, endocrine abnormalities, skin changes, splenomegaly, and thrombocytosis. Further investigations including serum immunofixation electrophoresis, VEGF measurement, and electromyography confirmed the diagnosis of POEMS syndrome. The diagnostic process spanned nearly 8 months, highlighting the syndrome’s atypical onset, diverse manifestations, and high risk of misdiagnosis. The details are shown in Table 1.

**Table 1:** Clinical timeline of the patient.

Date	Event/Examination/Treatment	Findings/Diagnostic significance
February 2024	Initial exacerbation of symptoms (dizziness, generalized edema, dyspnea)	Blood pressure 243/120 mmHg, suspected hypertensive emergency; symptoms improved after BP control
February 17, 2024	Hospitalization in Cardiology	Diagnosed with hypertension; diuretics relieved symptoms partially, but edema persisted
July 2024	Recurrent lower limb edema	Proteinuria 1+, serum albumin decreased to 36.4 g/L, suggesting renal insufficiency
September 8, 2024	Admitted to Gastroenterology for “ascites of unknown origin”	Lower limb ecchymosis, abdominal distension, significant mobility limitation; chief complaint of ascites first recorded
Early hospital stay	Comprehensive hepatic/renal function and ascites workup	Elevated creatinine (242 μmol/L), massive ascites; ruled out hepatic, tuberculous, malignant, and hypothyroid causes
Mid-September 2024	Abdominal paracentesis performed	Ascites non-infectious and non-tuberculous; elevated CA125 but no tumor cells; no definitive diagnosis yet
Late September 2024	Skin ulceration, worsening ecchymosis, increased mobility limitation	Skin lesions mimicking *Vibrio vulnificus* infection; infectious disease and dermatology teams suggested vascular etiology
Further assessment	Integrated findings: ascites, skin manifestations, hypothyroidism, splenomegaly, thrombocytosis	POEMS syndrome suspected
Early October 2024	Additional tests: EMG, serum immunofixation, VEGF, κ/λ ratio	Diagnostic criteria for POEMS met (neuropathy, monoclonal protein, elevated VEGF, etc.)
Final diagnosis	Joint consultation with Neurology and Hematology	Confirmed POEMS syndrome: IgA-lambda monoclonal gammopathy, VEGF 729.71 pg./ml, multisystem involvement

## Diagnostic assessment

The patient, a 65-year-old woman, was admitted with recurrent dizziness, generalized edema, abdominal distension, and progressive bilateral lower extremity ecchymosis. Initial workup revealed elevated platelet count, proteinuria, hypoalbuminemia, renal dysfunction, and exudative ascites, but no definitive evidence of infection (hemogram, CRP, PCT, and Pathogenic Microorganism Culture), malignancy (tumor marker, CT and MRI), autoimmune disease (ANAs, complement, and HIV), vasculitis (ANCA), or hypothyroidism-related serosal effusion (thyroid function). Cryoglobulin, antiphospholipid antibodies, and PCR testing for *V. vulnificus* in blister fluid were negative. Despite extensive evaluation, including thyroid ultrasound, tumor markers, abdominal imaging, and autoimmune serologies, the etiology of ascites remained unclear. Notably, she had significant skin changes resembling *V. vulnificus* infection, progressive symmetric sensorimotor neuropathy, hepatosplenomegaly, endocrine dysfunction (hypothyroidism), diffuse hyperpigmentation, pleural effusion, and thrombocytosis. These findings prompted consideration of POEMS syndrome. Subsequent tests confirmed elevated VEGF (729.71 pg./mL), IgA-λ type M-protein on serum immunofixation, abnormal serum free light chain ratio (*κ*/λ = 0.262), and electromyography showing polyneuropathy. A multidisciplinary consultation supported the diagnosis of POEMS syndrome.

## Therapeutic intervention

There is currently no standardized treatment regimen for POEMS syndrome, but therapeutic strategies are generally directed at eliminating the underlying plasma cell clone. Options include radiation for localized disease and systemic therapies—such as alkylator-based regimens, proteasome inhibitors, and autologous stem cell transplantation (ASCT)—for disseminated disease. ASCT is typically first-line, but due to the patient’s frailty and resource limitations, a bortezomib-based regimen was selected, which led to clinical improvement and normalization of VEGF levels. VEGF has been proposed as a useful biomarker for treatment response and disease monitoring, and its reduction in our case correlated well with symptom improvement. Following treatment, the patient’s condition stabilized, and she was discharged in a clinically improved state.

## Follow-up and outcomes

The patient was referred to the hematology department for further evaluation and treatment. After multiple discussions, the family consented to a bone marrow biopsy, which revealed *λ* light chain-restricted plasmacytosis, supporting the diagnosis of POEMS syndrome. She was initiated on a bortezomib-based chemotherapy regimen (bortezomib, cyclophosphamide, and dexamethasone), alongside supportive care including albumin supplementation, diuretics, and thyroid hormone replacement. Over the course of treatment, the patient showed gradual improvement in peripheral edema, ascites, and skin lesions. Neurological symptoms also stabilized, and repeat VEGF levels demonstrated a downward trend. After six cycles of chemotherapy, partial hematologic response was achieved with reduced M-protein levels and improved renal function. The patient remains under regular follow-up with plans for autologous stem cell transplantation upon achieving a deeper remission. However, following partial clinical improvement, the patient transferred to another institution for further management. Consequently, long-term follow-up data, including repeat electromyography, serum VEGF, and bone marrow reassessment, were not available. This limits the ability to comprehensively evaluate sustained response and neurological recovery. Regarding ASCT, we have clarified that the patient was initially unsuitable due to significant frailty, organ dysfunction, and socioeconomic factors. The option of ASCT was discussed during hematology consultations and remains a potential consideration at the referral center should a deeper remission be achieved.

## Discussion

POEMS syndrome is a rare but clinically significant plasma cell dyscrasia characterized by a constellation of multisystemic manifestations. Although the diagnosis requires the presence of both polyneuropathy and monoclonal plasma cell disorder, additional major and minor criteria are critical for diagnostic confirmation and disease monitoring. This case is particularly notable for its atypical dermatologic presentation-tense hemorrhagic bullae and violaceous ecchymoses-closely mimicking *V. vulnificus* skin infection. To our knowledge, such presentation has not been previously described in the literature, thereby expanding the known spectrum of cutaneous changes in POEMS syndrome.

Cutaneous manifestations are among the most prevalent and earliest features of POEMS syndrome, reported in up to 93% of patients, and may serve as critical diagnostic clues in otherwise obscure cases (3). These changes include hyperpigmentation, hemangiomas, hypertrichosis, white nails, skin thickening, and acrocyanosis (4). In this patient, the disease began with prominent purpuric plaques and lower limb edema, which initially mimicked *V. vulnificus* infection. The violaceous, painful, and infiltrative nature of the lesions, alongside systemic edema, created a misleading infectious appearance, delaying the consideration of a plasma cell disorder. This highlights the diagnostic challenge posed by atypical or exaggerated skin findings in POEMS, particularly when accompanied by overlapping systemic symptoms such as fluid overload or neuropathy.

In fact, skin changes often precede other diagnostic features such as neuropathy or monoclonal gammopathy, and are therefore of high diagnostic value. A retrospective analysis by Lee et al. (5) emphasized that patients with florid skin manifestations were more likely to be misdiagnosed initially with autoimmune or infectious conditions. While hemangiomas (glomeruloid or cherry-type) are considered characteristic, the presence of diffuse hyperpigmentation or violaceous plaques, especially in edematous regions, may be underrecognized. Moreover, the elevated VEGF levels in POEMS are thought to mediate increased vascular permeability and angiogenesis, which not only contribute to peripheral edema and ascites but may also explain the distinct vascular skin changes (6).

Therefore, clinicians should maintain a high index of suspicion for POEMS in patients presenting with unexplained or disproportionate skin findings, particularly when accompanied by signs of peripheral neuropathy, organomegaly, endocrinopathy, or monoclonal gammopathy. Early recognition of cutaneous manifestations can prompt appropriate investigations and expedite diagnosis, as in the present case, where skin lesions ultimately led to reevaluation and confirmation of POEMS syndrome.

The pathogenesis of POEMS-related skin changes remains incompletely understood but is thought to be largely driven by elevated levels of VEGF, a key pro-angiogenic cytokine secreted by clonal plasma cells (7). VEGF increases vascular permeability and induces endothelial dysfunction, potentially accounting for extravascular volume overload, papilledema, and cutaneous vascular changes (8). In this case, the extremely high VEGF concentration (729.7 pg./mL) likely contributed to capillary leakage, vascular fragility, and subsequent bullous transformation of ecchymoses. Similar mechanisms have been proposed in the formation of glomeruloid hemangiomas, a characteristic but not universally present finding in POEMS syndrome (9). The hemorrhagic and bullous lesions observed in our patient are highly unusual. They bear a striking resemblance to the rapid-onset, violaceous bullae seen in *V. vulnificus* septicemia, particularly in immunocompromised or cirrhotic individuals (10). Although the tense bullous lesions closely resembled those of *V. vulnificus* infection, the absence of relevant exposure history, negative microbiological and PCR results. Moreover, the bilateral, symmetrical, and non-infective distribution of lesions—along with other systemic features—prompted consideration of an alternative, the paraneoplastic nature of the skin findings.

The differential diagnosis in this patient was broad and included infectious cellulitis (particularly *V. vulnificus*), necrotizing fasciitis, vasculitis, cryoglobulinemia, and coagulopathies. However, the chronic progressive polyneuropathy, IgA-λ monoclonal band, organomegaly, endocrinopathy, and thrombocytosis fulfilled both mandatory and supporting criteria for POEMS syndrome (11). Notably, the presence of multiple system involvement often leads to extensive and delayed diagnostic workups. Awareness of rare dermatologic clues—such as the ones described here—may expedite recognition and management (12).

Although thyroid dysfunction was confirmed in our patient, gonadal hormone levels were not evaluated at the time of presentation, representing a limitation in fully characterizing the spectrum of endocrine involvement. In addition, although Castleman disease (CD) is an important major criterion in POEMS syndrome, no lymph node biopsy was performed due to patient and family refusal, and thus the presence of CD could not be confirmed or excluded in this case. Treatment of POEMS syndrome aims to eradicate the underlying plasma cell clone. ASCT is considered the first-line option in eligible patients and has shown excellent long-term outcomes. For patients who are frail or ineligible, bortezomib-based regimens, lenalidomide, or alkylating agents may provide clinical benefit. Our patient received six cycles of bortezomib-based therapy, with symptom improvement and VEGF reduction. While ASCT was considered for long-term disease control, the patient was referred to another center after six chemotherapy cycles and further follow-up data (including post-treatment photographs of the resolved skin lesions) are unavailable.

## Patient perspective

The patient expressed significant distress during the initial phase of her illness, particularly due to the progressive weakness, severe edema, and alarming skin changes that mimicked a severe infection. She described feelings of fear and uncertainty, especially when initial treatments showed limited response. However, after receiving a confirmed diagnosis and starting targeted therapy, she reported a sense of relief and hope. The gradual improvement in her symptoms, especially the reduction in skin lesions and the return of some mobility, greatly improved her confidence in the treatment. She expressed gratitude for the multidisciplinary team’s efforts and is now optimistic about her future treatment and recovery.

## Conclusion

This case highlights an unusual dermatologic presentation of POEMS syndrome characterized by rapidly evolving hemorrhagic bullae and ecchymoses that closely mimicked *V. vulnificus*–associated soft tissue infection. Such atypical skin lesions, although rare, may reflect underlying vascular fragility driven by elevated VEGF levels and should prompt consideration of a paraneoplastic etiology, particularly in the context of multisystem involvement. Early recognition, supported by serum VEGF measurement and immunofixation electrophoresis, is essential for timely diagnosis and initiation of appropriate therapy. This case expands the clinical spectrum of POEMS-associated skin manifestations and underscores the need for heightened awareness among clinicians when encountering unexplained vascular skin lesions in systemic disease contexts.

## Data Availability

The original contributions presented in the study are included in the article/supplementary material, further inquiries can be directed to the corresponding author.
